# Long-term real-world effectiveness and safety of fingolimod over 5 years in Germany

**DOI:** 10.1007/s00415-021-10931-w

**Published:** 2022-01-04

**Authors:** Tjalf Ziemssen, Michael Lang, Stephan Schmidt, Holger Albrecht, Luisa Klotz, Judith Haas, Christoph Lassek, Stefan Lang, Veronika E. Winkelmann, Benjamin Ettle, Ulf Schulze-Topphoff, the PANGAEA study group

**Affiliations:** 1 Center of Clinical Neuroscience, Neurological University Clinic Carl Gustav Carus, University of Technology, Dresden, Germany; 2 NeuroPoint Patient Academy and Neurological Practice, Ulm, Germany; 3 Health Center St. Johannes, Bonn, Germany; 4 Neurological Practice, Munich, Germany; 5 Department of Neurology, University Hospital Münster, Munster, Germany; 6 Center for Multiple Sclerosis, Jewish Hospital Berlin, Berlin, Germany; 7 Neurological Practice, Kassel and Vellmar, Vellmar, Germany; 8 Scientific and Medical Writing, Dormitz, Germany; 9 Novartis Pharma GmbH, Roonstr. 25, 90429 Nuremberg, Germany

**Keywords:** Relapsing–remitting multiple sclerosis, Fingolimod, Real-world, Safety, Effectiveness

## Abstract

**Objective:**

To evaluate the 5-year real-world benefit–risk profile of fingolimod in patients with relapsing–remitting MS (RRMS) in Germany.

**Methods:**

Post-Authorization Non-interventional German sAfety study of GilEnyA (PANGAEA) is a non-interventional real-world study to prospectively assess the effectiveness and safety of fingolimod in routine clinical practice in Germany. The follow-up period comprised 5 years. Patients were included if they had been diagnosed with RRMS and had been prescribed fingolimod as part of clinical routine. There were no exclusion criteria except the contraindications for fingolimod as defined in the European label. The effectiveness and safety analysis set comprised 4032 and 4067 RRMS patients, respectively.

**Results:**

At the time of the 5-year follow-up of PANGAEA, 66.57% of patients still continued fingolimod therapy. Annualized relapse rates decreased from baseline 1.5 ± 1.15 to 0.42 ± 0.734 at year 1 and 0.21 ± 0.483 at year 5, and the disability status remained stable, as demonstrated by the Expanded Disability Status Scale mean change from baseline (0.1 ± 2.51), the decrease of the Multiple Sclerosis Severity Score from 5.1 ± 2.59 at baseline to 3.9 ± 2.31 at the 60-months follow-up, and the percentage of patients with ‘no change’ in the Clinical Global Impression scale at the 60-months follow-up (78.11%). Adverse events (AE) occurring in 75.04% of patients were in line with the known safety profile of fingolimod and were mostly non-serious AE (33.62%) and non-serious adverse drug reactions (50.59%; serious AE 4.98%; serious ADR 10.82%).

**Conclusions:**

PANGAEA demonstrated the sustained beneficial effectiveness and safety of fingolimod in the long-term real-world treatment of patients with RRMS.

**Supplementary Information:**

The online version contains supplementary material available at 10.1007/s00415-021-10931-w.

## Introduction

Fingolimod (Gilenya, Novartis Pharma AG, Basel, Switzerland), an oral disease-modifying therapy (DMT) approved for relapsing–remitting multiple sclerosis (RRMS), has an extensive safety profile. In randomized clinical trials (RCTs), fingolimod demonstrated efficacy in reducing the frequency of relapses and disability progression in the long-term when compared to placebo [1–5].

Regulatory approval of fingolimod in Europe comprises the treatment of patients with highly active disease despite a full and adequate course of treatment with at least one disease-modifying therapy or patients with rapidly evolving severe relapsing remitting multiple sclerosis [6]. Obviously, the eligible population in clinical practice differs from the selected patients of RCTs, with respect to age, disease activity, comorbidities, concomitant medications, and prior MS treatment. Moreover, sample size and follow-up periods of RCTs limit both the generalizability of results to clinical practice and the probability of identifying rare therapy-related events [7, 8].

Therefore, the large prospective, 5-year Post-Authorization Non-interventional German sAfety study of GilEnyA (fingolimod) PANGAEA was initiated in 2011 [9]. Both a 12-months interim analysis [10] and 36-months follow-up of PANGAEA [11] showed the sustained effectiveness and safety of fingolimod, as demonstrated by reduced annualized relapse rates (ARRs) and stable Expanded Disability Status Scale (EDSS) scores, and the consistency of the frequency and nature of adverse events and adverse drug reactions.

Here, we report the results of the final 5-year follow-up of PANGAEA, a prospective, multi-center, non-interventional, long-term study on fingolimod. The main objective of this study was to assess the overall safety and effectiveness profile of fingolimod in more than 4000 RRMS patients treated by fingolimod under real-world-conditions in Germany.

## Methods

### Standard protocol approvals, registrations, and patient consents

An ethics committee approved the study before trial initiation and had jurisdiction over the medical director of the study. The study was conducted in accordance with the Declaration of Helsinki. Written informed consent was obtained from all participants before inclusion in PANGAEA.

### PANGAEA study design

PANGAEA is a prospective, multi-center, non-interventional, long-term study on fingolimod, conducted as part of routine clinical practice in Germany. PANGAEA was initiated in 2011 with the recruitment of patients. The overall study duration was from 4/2011 to 1/2019 and the observational period lasted a maximum of 5 years. The detailed study protocol has previously been published [9]. In brief, patients eligible for inclusion were required to have been diagnosed with RRMS [12], to have been prescribed fingolimod (0.5 mg) both as part of their routine clinical care and according to the approved German label of fingolimod, and to provide written informed consent. Prescription of fingolimod was solely based on the physician’s decision. The only exclusion criteria resulted from the contraindications defined in the European fingolimod summary of product characteristics (SmPC) [6].

### Data collection and study outcomes

Study follow-up visits took place every 3 months for a period of 60 months per patient, once the first month (first visit) was over, and data were recorded in standardized electronic case report forms. Effectiveness outcomes investigated and presented in this report were therapy continuation rates as well as reasons for premature treatment discontinuation and interruption, the number of relapses per patient (ARR), disability outcomes (EDSS scores) [13, 14], the proportions of patients with 6-month confirmed disability worsening or improvement, and the proportion of patients with no clinical disease activity. Confirmed disability worsening on EDSS was defined as 1.5-point worsening in patients with baseline scores = 0, as 1.0-point worsening in patients with baseline scores between 1.0 and 5.0, and as 0.5-point worsening in patients with baseline scores > 5.5 (confirmed disability improvement on EDSS was defined vice versa). EDSS changes had to be confirmed for at least 6 months. No disease activity was defined by the absence of both relapses and 6-month confirmed disability worsening. Furthermore, the Clinical Global Impression (CGI) scale [15] and the Multiple Sclerosis Severity Score (MSSS) [16] were assessed. For safety analyses, occurrence, duration, intensity, and outcome of adverse events (AEs) and serious AEs (SAEs) were documented throughout the observation period, regardless of their potential relation to treatment and irrespective of whether medication was taken as intended [17]. AEs and SAEs were classified using the Medical Dictionary for Regulatory Activities [18].

### Statistics

Statistical analyses were purely exploratory and descriptive. Categorical (nominal and ordinal) data were presented as absolute and relative frequencies. Relative frequencies were calculated based on all values including patients with missing data. Continuous data were categorized in clinically meaningful way and described by the mean ± SD and number of missing and non-missing values. To regard the effect of premature treatment discontinuation and documentation, data of all patients at their last completed visit were summarized as final follow-up (hereafter referred to as ‘last visit’). The exposure adjusted incidence rate (EAIR) of AEs has been defined as the number of patients with a specific event divided by the total follow-up time over all patients in years. Corresponding confidence intervals for incidence rates were calculated using the Clopper–Pearson formula. Statistical analyses were performed using the software package SAS release 9.4 TS1M3 (SAS Institute Inc., Cary, NC, USA).

### Data availability statement

In agreement with the consent forms signed by patients, subject-related data were transmitted and stored in pseudo-anonymized form and are therefore not publicly available. The study protocol has been published and is freely available [9].

## Results

### Study population and baseline characteristics

Out of 4206 patients enrolled, 4032 patients constituted the effectiveness analysis set comprising both patients who received fingolimod for the first time as part of PANGAEA (79.1%) and patients who had received fingolimod in previous trials before PANGAEA (20.9%) (see Supplementary Fig. 1). In PANGAEA, the mean duration of fingolimod treatment was 1192.4 ± 721.15 days within an average observational period of 1208.6 ± 720.62 days. The safety analysis encompassed 4067 patients who had received at least one dose of fingolimod.

Baseline characteristics of the effectiveness analysis set (*n* = 4032) are presented in Table 1. Patients were mostly female (71.92%) and most patients (> 90%) were between the age of 20–60 years. The most frequent MS diagnosis at study entry were relapsing remitting MS without or with mentioning of an acute exacerbation or progression and RRMS (G35.1-0 [53.03%], G35.1-1 [18.50%], G35.1 [15.10%]). On average, each patient had experienced 1.5 ± 1.15 relapses during the year before study enrollment, and most patients had EDSS scores ≤ 1.5 (22.99%), > 1.5 to ≤ 2.5 (21.70%), > 2.5 to ≤ 3.5 (13.74%), and > 3.5 to ≤ 4.5 (19.98%). Concerning MS-lesions, 84.30% of all patients had > 9 lesions in the T2 weighted scan or ≥ 1 gadolinium enhancing lesion at baseline. Almost one third of patients (32.19%, *n* = 1298) had any concomitant disease, but the proportions of patients with concomitant diseases such as diabetes mellitus (1.71%) chronic infections (0.10%), or renal dysfunction (0.05%) were low. A total of 28.29% and 41.39% of patients had prior (12 months) and concomitant non-MS treatment, respectively. Selective serotonin reuptake inhibitors were the most common prior (5.23%) and concomitant non-MS medication (8.11%). In the 3 years before study entry, 61.35% of patients had received any MS medication, most commonly interferon β-1A (22.86%) and glatiramer acetate (16.24%). During the study, approximately half of patients (55.23%) received concomitant medications for MS such as fampridine (12.69%), baclofen (8.53%), cholecalciferol (8.06%), and methylprednisolone (7.63%).

**Table 1:** Baseline characteristics of study participants (effectiveness analysis set)

	Effectiveness analysis set (*n* = 4032)
Demographic characteristics	
Gender (% females)	71.92% (male 28.03%)
Age at initial visit (years, mean ± SD)	39.1 ± 10.00
Body mass index (kg/m^2^, mean ± SD)	25.2 ± 5.24
MS diagnosis and history	
MS diagnosis at enrollment	
G35.1-0	53.03%
G35.1-1	18.50%
G35.1	15.10%
G35.9	9.35%
Other categories	< 2%
Time between MS diagnosis and start of PANGAEA (years, mean ± SD)	8.1 ± 6.27
Lesions	
Presence of contrast media enhancing lesions	37.48%
Multiple lesions in T2 weighted scan	74.13%
Absence of gadolinium enhancing lesions	55.26%
Disease activity	
Relapses per patient during the last 12 months (mean ± SD)	1.5 ± 1.15
EDSS	
≤ 1.5	22.99%
> 1.5 to ≤ 2.5	21.70%
> 2.5 to ≤ 3.5	13.74%
> 3.5 to ≤ 4.5	19.98%
> 4.5	13.71%
Not performed	0.6%
Missing	7.37%
Concomitant diseases	
Any concomitant disease	32.19%
Most commonly documented concomitant diseases	
Depression	6.17% (*n* = 249)
Hypertension	6.96% (*n* = 281)
Migraine	2.3% (*n* = 93)
Hypothyroidism	2.13% (*n* = 86)
Concomitant treatment	
Any concomitant non-MS treatment	41.39%
Any concomitant MS treatment	55.23%

*G35.1-0* relapsing remitting MS without mentioning of an acute exacerbation or progression, *G35.1-1* relapsing remitting MS with mentioning of an acute exacerbation or progression, *G35.1* RRMS, *G35.9* MS not otherwise specified, *EDSS* Expanded Disability Status Scale

### Effectiveness outcomes

#### Therapy interruption and discontinuation

During the 5-year follow-up, two thirds of 4032 patients stayed on fingolimod therapy (66.57%) and the majority of patients (87.38%) did not interrupt fingolimod treatment (one interruption in 10.76% of patients). Patients who prematurely discontinued fingolimod therapy (1348 of 4032 patients [33.43%]) primarily did so due to their own decision (30.59%), the occurrence of adverse events (22.56%), the switch to another physician (12.96%), and lack of effectiveness (5.83%; other reasons: Table 2). Throughout the study, the vast majority of physicians and patients (> 90% each; data not shown) rated tolerability as very good or good. Similar ratings of 84.15% (physicians) and 81.71% (patients) were received if analysis was restricted to the data obtained at the very last completed visit of each patient.

**Table 2:** Reasons for premature treatment discontinuation

Reasons for premature discontinuation	Number (%) of times a reason was given
Patient’s decision	598 (30.59%)
Adverse event	441 (22.56%)
Switch to another physician	248 (12.69%)
Disease progression/MS relapse	191 (9.77%)
Non-compliance	120 (6.14%)
Lack of effectiveness	114 (5.83%)
Lost to follow-up	108 (5.52%)
End of study	105 (5.37%)
Switch to other therapy	95 (4.86%)
Pregnancy/wish to become pregnant	83 (4.25%)
Switch to other study	59 (3.02%)
Physician’s decision	32 (1.64%)
Economic reasons	5 (0.26%)
Screening failure	4 (0.20%)
Other	63 (3.22%)
Missing	21 (1.07%)

#### Relapses

**Fig. 1:**
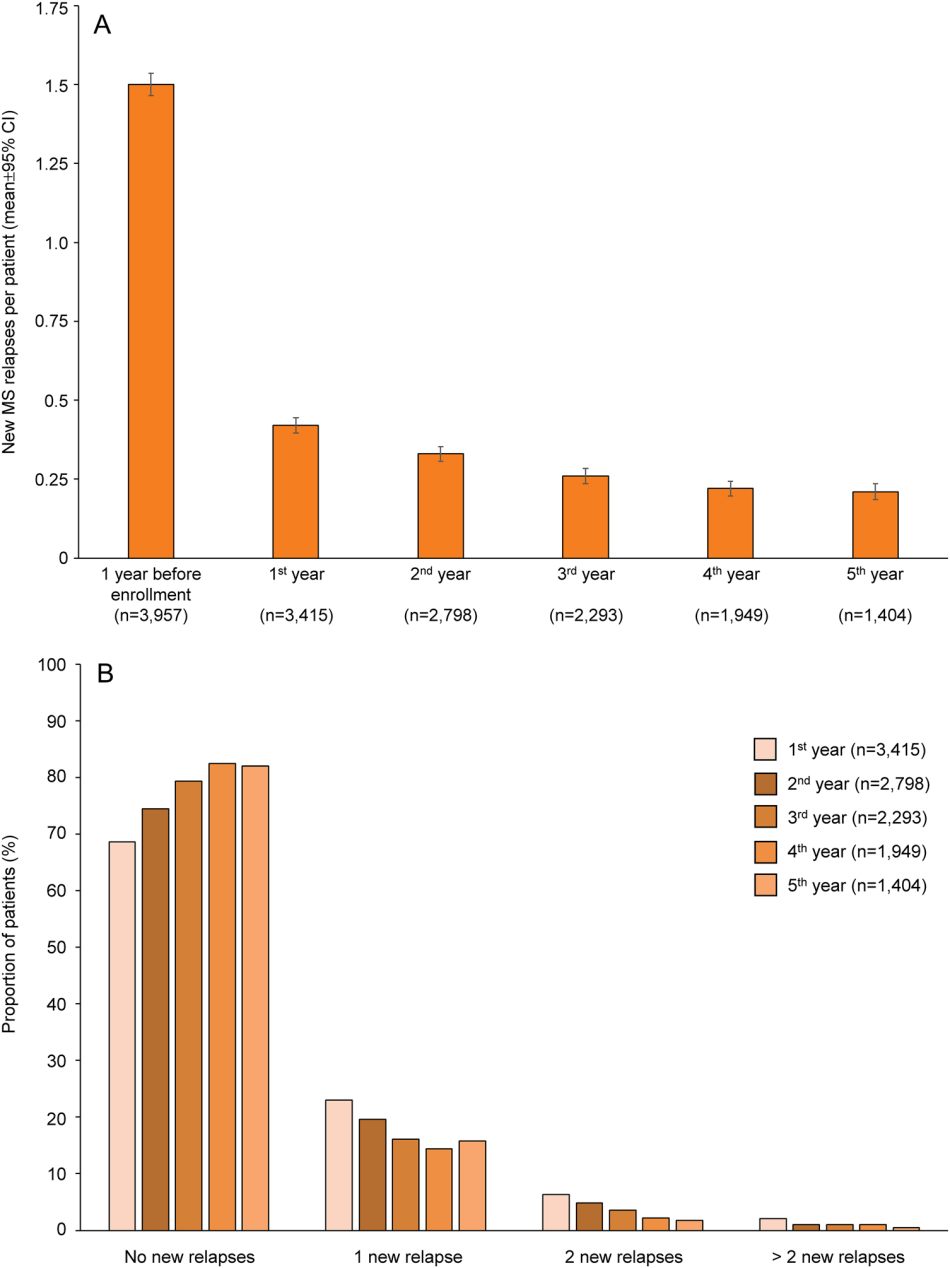
Relapse outcome during 5 years of fingolimod therapy. **A** New MS relapses per patient during the 1 year before fingolimod initiation and during each 1-year follow-up period after fingolimod initiation. Data are presented as mean ± 95% CI. **B** Proportion of patients with no, one, two, and more than two new relapses during 5 years of follow-up

The number of new MS relapses per patient continuously decreased each study year, from 1.5 ± 1.15 before the start of PANGAEA (*n* = 3957) to 0.42 ± 0.734 at year 1 (*n* = 3415) and 0.21 ± 0.483 at year 5 (*n* = 1404; Fig. 1A). During the 5 years of PANGAEA, the proportion of patients who were free of relapses was between 68.61 and 82.45% (Fig. 1B). In the 1799 patients who experienced new relapses during PANGAEA, most relapses did not require hospitalization (77.04%) and were of moderate intensity (moderate: 63.42%; mild: 26.4%; severe 9.95%). Outcome of MS relapses were mostly complete remission (26.68%) and extensive remission (35.46%).

#### Disability progression

**Fig. 2:**
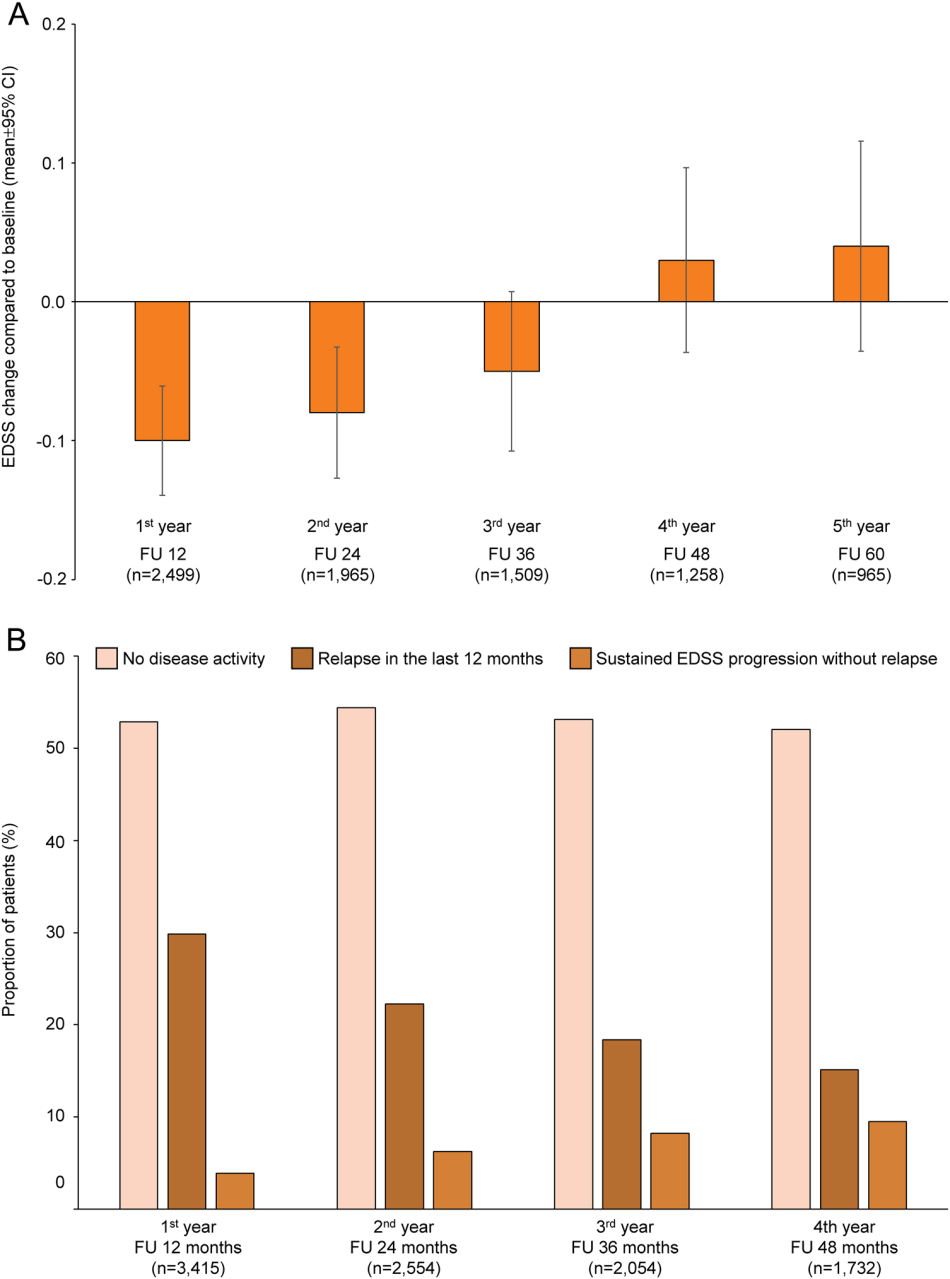
Disability outcome during 5 years of fingolimod therapy. **A** Mean EDSS change during the 60 months of PANGAEA (mean ± 95% CI). Data of the follow-up visits at month 12, 24, 36, 48, and 60 are presented. **B** Proportions of patients who had no clinical disease activity, experienced relapses during the last 12 months, and showed sustained 6-month-confirmed EDSS progression without the detection of relapses. Since EDSS progression requires conformation at two or more visits separated by 6 months, no assessment at 60 months can be provided due to end of study period (*EDSS* Expanded Disability Status Scale; *FU* follow-up visit)

The mean EDSS score at baseline was 3.0 ± 1.68 (*n* = 3711) and did not substantially change throughout the study (Fig. 2A). From follow-up visit at month 3 (*n* = 3690) to month 54 (*n* = 1537), the proportion of patients with 6-month confirmed disability improvement and confirmed disability worsening increased from 6.69% and 3.50% to 10.54% and 11.06%, respectively (stable EDSS at 54-months-follow-up: 49.64%; missing data 28.76%). At the follow-up visits after 12, 24, 36, and 48 months, more than half of patients showed no clinical disease activity, as defined by the absence of both relapses and 6-month confirmed disability worsening in the last 12 months (since EDSS progression needs to be confirmed over 6 months, no 60 months assessment is provided due to end of study period; Fig. 2B).

Last visit data indicated stability of disability levels in most patients. The EDSS score at the last completed visit of 2964 patients was 3.1 ± 1.89 (baseline 3.0 ± 1.68, *n* = 3711). Adjusting disability for disease duration using the MSSS algorithm also demonstrated constant values at the last visit (4.4 ± 2.58, *n* = 2791) when compared to baseline (5.1 ± 2.59, *n* = 3503). The MSSS score at the 60-months follow-up was 3.9 ± 2.31 (*n* = 966). Likewise, the CGI improvement scale revealed ‘no change’ in 64.27% of 3960 patients at the last visit (very much improved 0.48%, much improved 3.28%, minimally improved 5.83%, minimally worse 12.35%, much worse 2.27%, very much worse 0.18%) and 78.11% of 1334 patients at the 60-months follow-up.

### Safety outcomes

The safety analysis set included all patients who had received at least one dose of fingolimod (*n* = 4067). Of these, 3052 patients (75.04%) experienced a total of 15,939 AEs during the study (no AE in 1015 patients [24.96%]). These AEs were categorized as non-serious AE (33.62%), serious AE (SAE 4.98%), non-serious adverse drug reactions (nsADR 50.59%), and serious ADR (SADR 10.82%). The most frequently reported outcome of all events was ‘recovered’ (60.98%; ‘not yet recovered’: 28.16%), and 865 patients (5.43%) required hospitalization. There were 18 fatal outcomes.

Regardless of seriousness and relatedness to therapy of AEs, the most frequently reported AEs were infections (32.78%; most frequently nasopharyngitis [15.44% (95% confidence interval [CI] 14.34–16.59%)]), abnormal laboratory findings (30.12%; most frequently: increased gamma-glutamyltransferase [6.88% (95% CI 6.12–7.71%] and decreased lymphocyte counts [6.47% (95% CI 5.73–7.27%)]), nervous system disorders (26.73%; most frequently: MS relapse [7.08% (95% CI 6.31–7.92%)]), and blood and lymphatic system disorders (17.14%; most frequently lymphopenia [11.73% (95% CI 10.75–12.76%] and leukopenia [7.16% (95% CI 6.38–8.00%)]) (Supplementary Table 1).

Specific events that had occurred during the first 12 months of PANGAEA [11] were defined as adverse event of special interest (AESI) that are of particular scientific and medical relevance and require continuous monitoring (Table 3). The most frequently occurring AESI were hypertension (*n* = 255 [6.27%]), lymphopenia (*n* = 477 [11.73%]), leukopenia (*n* = 291 (7.16%]), and increased hepatic enzyme levels (*n* = 230 [5.66%]).

**Table 3:** Adverse events of special interest (AESI) detected in the safety analysis set (*n* = 4067)

System	Preferred term	*N* (%)	EAIR per year (95% CI)
Cardiac events	Hypertension	255 (6.27%)	0.021 [0.018, 0.023]
Infections	Herpes zoster	122 (3.0%)	0.010 [0.008, 0.011]
	Progressive multifocal leukoencephalopathy	2 (0.05%)	0.000 [0.000, 0.001]
	Meningitis, cryptococcal	1 (0.02%)	0.000 [0.000, 0.000]
Leukopenia	Lymphopenia	477 (11.73%)	0.040 [0.037, 0.044]
	Leukopenia	291 (7.16%)	0.024 [0.021, 0.027]
	White blood cell count decreased	115 (2.83%)	0.009 [0.007, 0.011]
Diseases of the nervous system	Posterior reversible encephalopathy syndrome	0	0.000 [0.000, 0.000]
	Acute disseminated encephalomyelitis	0	0.000 [0.000, 0.000]
Hepatic enzymes	Hepatic enzyme level increased	230 (5.66%)	0.018 [0.016, 0.021]
	Alanine aminotransferase level increased	187 (4.60%)	0.015 [0.013, 0.017]
Eye disorder	Macular edema	20 (0.49%)	0.002 [0.001, 0.002]
Neoplasms	Thyroid cancer	1 (0.02%)	0.000 [0.000, 0.000]
	Benign breast neoplasm	1 (0.02%)	0.000 [0.000, 0.000]
	Benign neoplasm	1 (0.02%)	0.000 [0.000, 0.000]
Lymphoma	Diffuse large B-cell lymphoma stage I	1 (0.02%)	0.000 [0.000, 0.000]
	Follicle center lymphoma, follicular grade I, II, III stage IV	1 (0.02%)	0.000 [0.000, 0.000]
	Non-Hodgkin’s lymphoma	1 (0.02%)	0.000 [0.000, 0.000]
Pregnancy	Abortion spontaneous	7 (0.17%)	0.001 [0.000, 0.001]
	Abortion	3 (0.07%)	0.000 [0.000, 0.001]
	Abortion incomplete	1 (0.02%)	0.000 [0.000, 0.000]

*EAIR* exposure adjusted incidence rate

PANGAEA further investigated the occurrence of rare adverse events that have been published in case reports. Cryptococcal meningitis and progressive multifocal leukoencephalopathy (PML) were detected in one patient (0.02% [95% CI 0.00–0.14%]) and two patients (0.05% [95% CI 0.00–0.18%]), respectively. PML causing JC-polyomavirus was detected in two patients (0.05% [95% CI 0.00–0.18%]). Thrombocytopenia occurred in 10 patients (0.25% [95% CI 0.11–0.46%]). Liver function tests performed in 84.10% of patients revealed relevant diagnostic findings in 1.42%, and ophthalmological examinations performed in 52.65% of patients identified current or anamnestic macular edema in 0.07% of patients. Elevated levels of alanine aminotransferase were found in 187 out of 4067 patients (4.60% [95% CI 3.97–5.29%]).

## Discussion

Here, we report the results of the 5-year follow-up of PANGAEA, a non-interventional, long-term study to assess effectiveness and safety of fingolimod in 4000 RRMS patients treated under real-world-conditions in Germany. In PANGAEA, fingolimod markedly reduced ARRs and stabilized EDSS scores in the long term. The majority of fingolimod treated patients showed no clinical disease activity during the 5-year follow-up. Overall, this long-term study confirmed the positive benefit-risk profile of fingolimod demonstrated in both RCTs and other real-world studies.

As a non-interventional real-world study, PANGAEA complements and expands the data obtained in RCTs. The average observation and treatment period of PANGAEA as well as its large sample size by far exceeds those of RCTs on fingolimod [2, 4, 5]. Both age and female gender of RRMS patients in PANGAEA corresponded to those found in a large German register study on 13,333 MS patients [19]. Patients who entered PANGAEA had similar mean ages and disease durations but more active disease at baseline when compared to patients participating in RCTs on fingolimod. Patients in PANGAEA presented with a greater variety of concomitant therapies and comorbid conditions than patients in RCTs [2, 4, 10].

The sustained reduction of relapses by fingolimod in PANGAEA corresponds to that observed in both RCTs [2, 4] and recent retrospective real-world studies [20, 21]. A similar but less pronounced long-term reduction of relapses was observed with other treatments [22]. During the 5 years of PANGAEA, both the mean EDSS scores as well as the proportion of patients with no clinical disease activity (defined as no relapses and no 6-month confirmed disability progression) remained stable in patients on drug, which is an important confirmation of the results of other real-world studies and shorter follow-up analyses [11, 20].

Treatment adherence and compliance, especially in chronic diseases such as MS, are crucial for clinical benefit [23]. In PANGAEA, two thirds of patients continued fingolimod therapy in the long term, which lays within the range of adherence rates to other chronic medications [24], but was lower than that observed in RCTs [2, 4] and real-world studies on fingolimod with shorter follow-up periods [11, 20, 21]. It has been demonstrated that persistence with and adherence to oral MS medications such as fingolimod are generally higher than those to injectable and infusible DMTs [25]. However, several factors have been associated with reduced treatment adherence [26], including cognitive impairment, duration of disease and treatment [27], personality traits [28], and AEs [24]. Indeed, the major reasons for treatment discontinuation in PANGAEA were the patient’s decision and AEs, which is consistent with other real-world studies of fingolimod [20, 29]. Comorbidities and concomitant medications may predispose the populations of real-world studies to specific AEs, which might be only observed during long-term follow-up periods [3, 30, 31]. Safety issues that have been identified during the clinical development of fingolimod such as the occurrence of bradycardia and other cardiac events did not require additional safety considerations in PANGAEA. Increased levels of gamma-glutamyltransferase and alanine aminotransferase as well as decreased lymphocyte counts were as expected [2, 4, 11, 20, 32]. Rare adverse events of fingolimod that have occasionally been described in case reports such as fingolimod-associated PML [33], cryptococcal meningoencephalitis [34], and thrombocytopenia [35] occurred with very low frequency and did not raise new safety concerns. To conclude, this 5-year follow-up of PANGAEA confirmed the known and manageable safety profile of fingolimod but the identification of specific AEs potentially affecting treatment adherence might further increase the clinical benefit of fingolimod [36].

A strength of PANGAEA is that it analyzed an extensive amount of data on patients receiving fingolimod in accordance with the fingolimod SmPC [6] and as part of routine clinical practice at neurologic centers across Germany. In contrast to other real-world studies and RCTs, PANGAEA collected data for a longer period and systematically assessed comorbidities and concomitant medication, thereby expanding the known safety profile of fingolimod.

We recognized the following three limitations of our study. First, effectiveness and safety of fingolimod was assessed in a German population of RRMS patients treated in accordance with the European treatment label [6]. Therefore, these results cannot be generalized for other countries without constraints. Second, patients were enrolled in a consecutive order to minimize selection bias, but other biases inherent to observational studies on unselected populations might have been occurred [37]. However, even if real-world studies cannot provide the same level of evidence as RCTs, PANGAEA still provides important information on the long-term clinical benefits and the occurrence of rare adverse events not captured by RCTs. Third, 21.9% of patients enrolled in PANGAEA were already enrolled in previous fingolimod trials, which has to be taken into account when interpreting treatment effects.

In conclusion, the 5-year follow-up of PANGAEA confirmed the well-established benefit-risk profile of fingolimod in long-term clinical practice which is key for strategic MS treatment [38]. It investigated a broad and heterogenous spectrum of real-world RRMS patients, thereby complementing and expanding the efficacy and safety data on fingolimod obtained in RCTs.

## Supplementary Information

Below is the link to the electronic supplementary material.

Supplementary file1 (DOCX 45 kb)

## Data Availability

Data is available upon reasonable request to the study sponsor.
