# Supplementary material for: Long-term real-world effectiveness and safety of fingolimod over 5 years in Germany
Source: J Neurol. 2022 Jan 4;269(6):3276–85. doi: 10.1007/s00415-021-10931-w (PMC9120082; doi:10.1007/s00415-021-10931-w)

Supplementary Table 1 Confidence intervals and EAIR for all documented AEs by MedDRA primary SOCs and Preferred Terms

Population: Safety Analysis Set (N=4067)

| System | Preferred term | N (%) | EAIR per year (95% CI)^1)^ |
| --- | --- | --- | --- |
| Infections and infestations | Nasopharyngitis | 628 (15.44%) | 0.055 [0.050, 0.059] |
|  | Urinary tract infection | 163 (4.01%) | 0.013 [0.011, 0.015] |
|  | Bronchitis | 155 (3.81%) | 0.012 [0.010, 0.014] |
|  | Herpes zoster | 122 (3.00%) | 0.010 [0.008, 0.011] |
| Investigations | Gamma-glutamyltransferase increased | 280 (6.88%) | 0.023 [0.020, 0.026] |
|  | Lymphocyte count decreased | 263 (6.47%) | 0.021 [0.019, 0.024] |
|  | Hepatic enzyme increased  Low density lipoprotein increased | 230 (5.66%)  228 (5.61%) | 0.018 [0.016, 0.021]  0.019 [0.016, 0.021] |
| Nervous system disorders | Multiple sclerosis relapse | 288 (7.08%) | 0.022 [0.019, 0.024] |
|  | Headache | 226 (5.56%) | 0.018 [0.016, 0.021] |
|  | Dizziness | 152 (3.74%) | 0.012 [0.010, 0.014] |
|  | Migraine | 84 (2.07%) | 0.007 [0.005, 0.008] |
| Blood and lymphatic system disorders | Lymphopenia | 477 (11.73%) | 0.040 [0.037, 0.044] |
|  | Leukopenia  Monocytosis  Lymphadenopathy | 291 (7.16%)  26 (0.64%)  21 (0.52%) | 0.024 [0.021, 0.027]  0.002 [0.001, 0.003]  0.002 [0.001, 0.002] |
| General disorders and administration site conditions | Fatigue | 209 (5.14%) | 0.017 [0.014, 0.019] |
|  | Pyrexia | 70 (1.72%) | 0.005 [0.004, 0.007] |
|  | Gait disturbance | 69 (1.70%) | 0.005 [0.004, 0.007] |
|  | Pain | 56 (1.38%) | 0.004 [0.003, 0.006] |
| Musculoskeletal and connective tissue disorders | Back pain | 123 (3.02%) | 0.010 [0.008, 0.012] |
|  | Pain in extremity | 100 (2.46%) | 0.008 [0.006, 0.010] |
|  | Arthralgia | 77 (1.89%) | 0.006 [0.005, 0.008] |
|  | Muscle spasms | 36 (0.89%) | 0.003 [0.002, 0.004] |
| Psychiatric disorders | Depression | 172 (4.23%) | 0.014 [0.012, 0.016] |
|  | Sleep disorder | 98 (2.41%) | 0.008 [0.006, 0.009] |
|  | Depressed mood | 47 (1.16%) | 0.004 [0.003, 0.005] |
|  | Panic attack | 31 (0.76%) | 0.002 [0.002, 0.003] |
| Gastrointestinal disorders | Nausea | 105 (2.58%) | 0.008 [0.007, 0.010] |
|  | Diarrhoea | 103 (2.53%) | 0.008 [0.007, 0.010] |
|  | Abdominal pain upper | 63 (1.55%) | 0.005 [0.004, 0.006] |
|  | Vomitting | 35 (0.86%) | 0.003 [0.002, 0.004] |
| Skin and subcutaneous tissue disorders | Alopecia | 83 (2.04%) | 0.006 [0.005, 0.008] |
|  | Rash | 62 (1.52%) | 0.005 [0.004, 0.006] |
|  | Pruritus | 30 (0.74%) | 0.002 [0.002, 0.003] |
|  | Eczema | 25 (0.61%) | 0.002 [0.001, 0.003] |
| Vascular disorders | Hypertension | 255 (6.27%) | 0.021 [0.018, 0.023] |
|  | Haematoma | 14 (0.34%) | 0.001 [0.001, 0.002] |
|  | Hypertensive crisis | 11 (0.27%) | 0.001 [0.001, 0.002] |
|  | Hypotension | 10 (0.25%) | 0.001 [0.000, 0.001] |
| Metabolism and nutrition disorders | Vitamin D deficiency | 210 (5.16%) | 0.017 [0.015, 0.019] |
|  | Hypercholesterolaemia | 31 (0.76%) | 0.002 [0.002, 0.003] |
|  | Iron deficiency | 16 (0.39%) | 0.001 [0.001, 0.002] |
|  | Vitamin B12 deficiency | 16 (0.39%) | 0.001 [0.001, 0.002] |
| Respiratory, thoracic and mediastinal disorders | Cough | 122 (3.00%) | 0.010 [0.008, 0.011] |
|  | Oropharyngeal pain | 58 (1.43%) | 0.005 [0.003, 0.006] |
|  | Dyspnoea | 31 (0.76%) | 0.002 [0.002, 0.003] |
|  | Epistaxis | 11 (0.27%) | 0.001 [0.000, 0.002] |
| Injury, poisoning and procedural complications | Fall | 102 (2.51%) | 0.008 [0.007, 0.010] |
|  | Contusion | 24 (0.59%) | 0.002 [0.001, 0.003] |
|  | Road traffic accident | 14 (0.34%) | 0.001 [0.001, 0.002] |
|  | Foot fracture | 13 (0.32%) | 0.001 [0.001, 0.002] |
| Eye disorders | Visual impairment | 64 (1.57%) | 0.005 [0.004, 0.006] |
|  | Vision blurred | 26 (0.64%) | 0.002 [0.001, 0.003] |
|  | Macular oedema | 20 (0.49%) | 0.002 [0.001, 0.002] |
|  | Glaucoma | 19 (0.47%) | 0.001 [0.001, 0.002] |
| Cardiac disorders | Bradycardia | 38 (0.93%) | 0.003 [0.002, 0.004] |
|  | Palpitations | 30 (0.74%) | 0.002 [0.002, 0.003] |
|  | Tachycardia | 27 (0.66%) | 0.002 [0.001, 0.003] |
|  | Atrioventricular block second degree | 19 (0.47%) | 0.001 [0.001, 0.002] |
| Neoplasms benign, malignant and unspecified (incl cysts and polyps) | Basal cell carcinoma | 25 (0.61%) | 0.002 [0.001, 0.003] |
|  | Melanocytic naevus | 20 (0.49%) | 0.002 [0.001, 0.002] |
|  | Skin papilloma | 13 (0.32%) | 0.001 [0.001, 0.002] |
|  | Breast cancer | 8 (0.20%) | 0.001 [0.000, 0.001] |
| Renal and urinary disorders | Urge incontinence | 21 (0.52%) | 0.002 [0.001, 0.002] |
|  | Urinary retention | 20 (0.49%) | 0.002 [0.001, 0.002] |
|  | Micturition urgency | 19 (0.47%) | 0.001 [0.001, 0.002] |
|  | Bladder disorder | 13 (0.32%) | 0.001 [0.001, 0.002] |
| Ear and labyrinth disorders | Vertigo | 35 (0.86%) | 0.003 [0.002, 0.004] |
|  | Sudden hearing loss | 14 (0.34%) | 0.001 [0.001, 0.002] |
|  | Tinnitus | 14 (0.34%) | 0.001 [0.001, 0.002] |
|  | Vertigo positional | 12 (0.30%) | 0.001 [0.000, 0.002] |
| Surgical and medical procedures | Wisdom teeth removal | 7 (0.17%) | 0.001 [0.000, 0.001] |
|  | Abortion induced | 6 (0.15%) | 0.000 [0.000, 0.001] |
|  | Hysterectomy | 5 (0.12%) | 0.000 [0.000, 0.001] |
|  | Mole excision | 5 (0.12%) | 0.000 [0.000, 0.001] |
| Reproductive system and breast disorders | Erectile dysfunction | 12 (0.30%) | 0.001 [0.000, 0.002] |
|  | Cervical dysplasia | 11 (0.27%) | 0.001 [0.000, 0.002] |
|  | Menorrhagia | 7 (0.17%) | 0.001 [0.000, 0.001] |
|  | Amenorrhoea | 4 (0.10%) | 0.000 [0.000, 0.001] |
| Hepatobiliary disorders | Cholelithiasis | 16 (0.39%) | 0.001 [0.001, 0.002] |
|  | Cholecystitis | 7 (0.17%) | 0.001 [0.000, 0.001] |
|  | Hyperbilirubinaemia | 6 (0.15%) | 0.000 [0.000, 0.001] |
|  | Hepatic steatosis | 4 (0.10%) | 0.000 [0.000, 0.001] |
| Immune system disorders | Hypersensitivity | 13 (0.32%) | 0.001 [0.001, 0.002] |
|  | Seasonal allergy | 10 (0.25%) | 0.001 [0.000, 0.001] |
|  | Drug hypersensitivity | 4 (0.10%) | 0.000 [0.000, 0.001] |
|  | Allergy to animal | 2 (0.05%) | 0.000 [0.000, 0.001] |
| Endocrine disorders | Hypothyroidism | 8 (0.20%) | 0.001 [0.000, 0.001] |
|  | Thyroid mass | 7 (0.17%) | 0.001 [0.000, 0.001] |
|  | Goitre | 4 (0.10%) | 0.000 [0.000, 0.001] |
|  | Hyperthyroidism | 3 (0.07%) | 0.000 [0.000, 0.001] |
| Congenital, familial and genetic disorders | LUMBAR syndrome | 2 (0.05%) | 0.000 [0.000, 0.001] |
|  | Asplenia | 1 (0.02%) | 0.000 [0.000, 0.000] |
|  | Atrial septal defect | 1 (0.02%) | 0.000 [0.000, 0.000] |
|  | Congenital hydronephrosis | 1 (0.02%) | 0.000 [0.000, 0.000] |
| Pregnancy, puerperium and perinatal conditions | Abortion spontaneous | 7 (0.17%) | 0.001 [0.000, 0.001] |
|  | Abortion | 3 (0.07%) | 0.000 [0.000, 0.001] |
|  | Abortion incomplete | 1 (0.02%) | 0.000 [0.000, 0.000] |
|  | Ectopic pregnancy | 1 (0.02%) | 0.000 [0.000, 0.000] |
| Social circumstances | Stress at work | 2 (0.05%) | 0.000 [0.000, 0.001] |
|  | Ear piercing | 1 (0.02%) | 0.000 [0.000, 0.000] |
|  | Ex-tobacco user | 1 (0.02%) | 0.000 [0.000, 0.000] |

^1)^ EAIR: exposure adjusted incidence rate.

**Supplementary Figure 1 CONSORT Diagram**

CONSORT Diagram showing patient flow of the PANGAEA study.


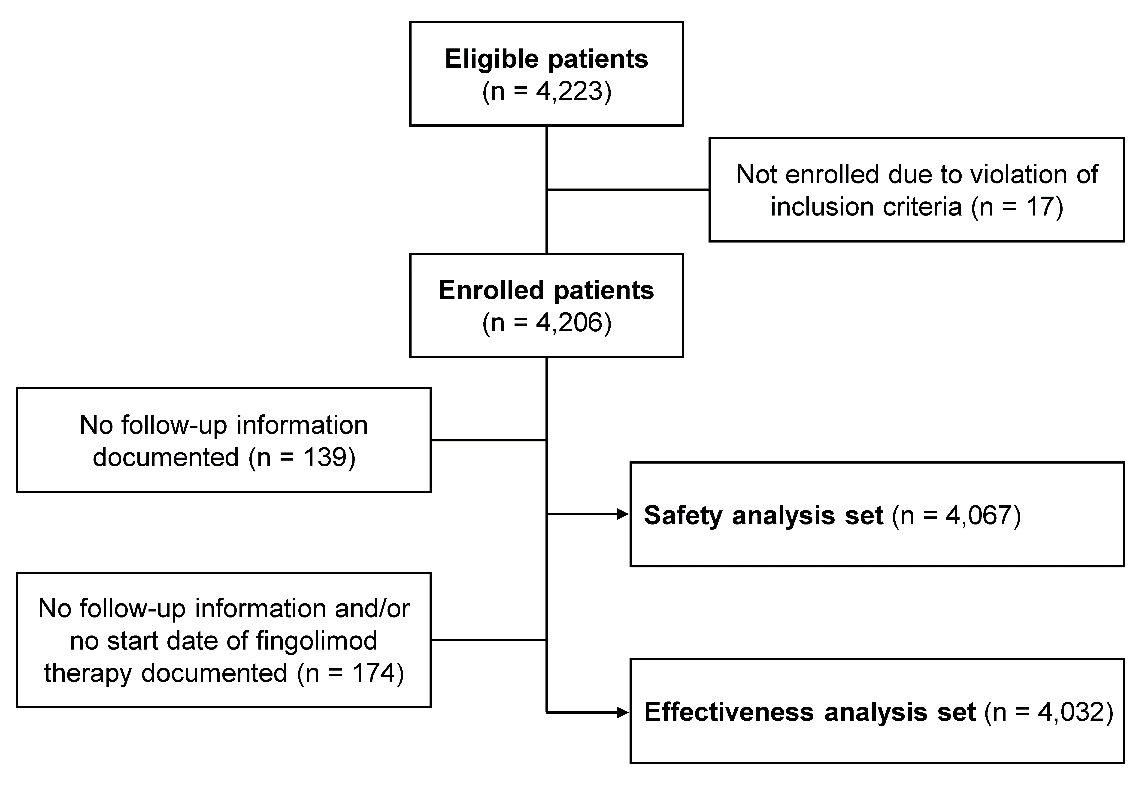

Supplement: Supplementary file 1 — Supplementary file1 (DOCX 45 kb) [file 415_2021_10931_MOESM1_ESM.docx]
